# Digitalization through the use of Ren’Py-Based interactive learning experiences in physiotherapy and rehabilitation education: a randomised, controlled, single-blind study

**DOI:** 10.1186/s12909-025-08474-z

**Published:** 2025-12-22

**Authors:** Ahmet Koçyiğit, Uğur Cavlak

**Affiliations:** 1 Department of Physiotherapy and Rehabilitation, Biruni University, İstanbul, Turkey; 2 Department of Physiotherapy and Rehabilitation, Zonguldak Bülent Ecevit University, Zonguldak, Turkey

**Keywords:** Digitalization, Digital classroom, Face-to-face education, Physiotherapy education

## Abstract

**Background:**

Advances in health professions education increasingly emphasize the use of digital technologies to enhance student engagement and support diverse learning needs. In physiotherapy training, particularly in technically complex subjects like electrotherapy, conventional instruction may fall short in fostering active learning and knowledge retention. Game-based platforms such as Ren’Py offer an opportunity to integrate interactive, scenario-based learning into the curriculum. This study aimed to assess the impact of Ren’Py-based digital materials on learning outcomes by comparing conventional, digital, and hybrid teaching models in an undergraduate electrotherapy course.

**Methods:**

This single-blind, three-arm randomized controlled trial was conducted between October-December 2024 at the Department of Physiotherapy and Rehabilitation. Eighty second-year physiotherapy students who had not previously taken the course were randomly assigned via computerized sequence to one of three groups: Conventional Education Group (CEG, *n* = 23), Digital Education Group (DEG, *n* = 29), or Conventional and Digital Education Group (CaDEG, *n* = 28). Instructional delivery included theoretical and practical sessions, followed by theoretical and practical exams. Outcomes included exam scores, the Cognitive Load Scale, the Attitude Scale Towards the Physiotherapy Profession, and the Educational Materials Motivation Survey. The data were analyzed using SPSS 27.0 package program. The parametric ANOVA test was used for normally distributed data, and the nonparametric Kruskal-Wallis test was used for nonnormally distributed data. Post-hoc analyses were performed using the Bonferroni test for normally distributed data and the Dunn-Bonferroni test for non-normally distributed data. A *p* < 0.05 level of significance was accepted for all findings.

**Results:**

Theoretical exam scores were similar between groups (CI(95%) = 0.00 to 0.07; *p* = 0.616), but practical exam scores were significantly different (CI(95%) = 0.03 to 0.30; *p* < 0.001); both CEG and CaDEG performed better than DEG (CI(95%) = 2.22 to 18.01, *p* = 0.007 and CI(95%)=-18.51 to -3.52, *p* = 0.002, respectively). In ASTPP scores, no significant difference was observed between the groups in pre-training, post-training, post-pre-training differences, and in within-group comparisons in any of the CEG, DEG and CaDEG groups (CI(95%) = 130.13 to 136.16, *p* = 0.858; CI(95%) = 131.91 to 137.08, *p* = 0.511; CI(95%)=-1.27 to 3.97, *p* = 0.852; CI(95%)=-4.59 to 3.63, *p* = 0.987; CI(95%)=-7.31 to 5.10, *p* = 0.335; CI(95%)=-5.22 to 0.57, *p* = 0.075, respectively). Cognitive load was significantly higher in the CEG group compared to DEG and CaDEG (CI(95%)=-2.04 to 0.09, *p* = 0.020; CI(95%)=-0.01 to 2.11, *p* = 0.016, respectively). IMMS scores were significantly higher in the CaDEG group than in CEG (CI(95%) = 1.77 to 30.32, *p* = 0.022).

**Conclusion:**

Face-to-face and hybrid models were more effective than digital-only instruction for developing practical skills in physiotherapy education. The hybrid model also reduced cognitive load and increased motivation. These findings suggest that integrating tools like Ren’Py into conventional instruction may enhance learning when used as a complement. Further studies with larger samples and extended durations are recommended.

**Trial registration:**

This study was retrospectively registered on ClinicalTrials.gov (NCT07274839).

**Supplementary Information:**

The online version contains supplementary material available at 10.1186/s12909-025-08474-z.

## Background

The increasing digitalization of education has led to the widespread adoption of internet-based tools that facilitate access to information independent of time and location. Particularly in disciplines such as the health sciences, where knowledge acquisition and skill development are both essential, educational technologies are being leveraged to enhance learning outcomes and student engagement. As higher education institutions operate within an environment of rapid technological evolution, both students and educators are expected to continuously adapt their practices to remain effective and relevant [1].

Effective use of digital educational tools has the potential to increase student engagement as well as provide a more effective learning experience. This is supported by many studies that highlight the significant benefits of technology integration in education. For example, Freeman et al. [2] found that combining active learning strategies with digital tools significantly increased student engagement and improved learning achievement. Similarly, Anderson & Dron [3] reported that online learning environments encouraged active student participation and provided more effective learning outcomes than more conventional educational methods.

Physiotherapy education requires continuous development to adapt to new processes created by globalization, technological developments, and economic changes [4]. In this process, education providers are developing new pedagogical strategies and adopting and implementing various models such as competency-based learning and online technologies [5]. Recent studies have reported that digital tools in physiotherapy education can improve accessibility, student autonomy, and engagement, but may fall short in supporting the development of hands-on clinical competencies without complementary in-person practice [6]. Physiotherapy curricula are shaped by the combination of theoretical knowledge, skill training, and practice [7]. In Physiotherapy and Rehabilitation education, it is important to teach practical skills as well as theoretical knowledge effectively. Conventional education methods are usually limited to classroom lectures and laboratory practices. However, the integration of digital education materials can provide students with richer and more interactive learning experiences [8]. Interactive websites and applications provide students with flexibility, ease of access, and openness, providing them with the opportunity to both learn theoretical knowledge and observe how practical skills are applied [9]. The need for online and interactive learning tools, especially after the COVID-19 pandemic, has made the importance of innovative solutions in physiotherapy education even more evident [10]. Recent studies have explored various digital education tools in physiotherapy education, including video-based instruction, learning management systems, and simulation technologies, all of which aim to enhance flexibility, engagement, and skill acquisition [9].

Despite growing interest in digital tools, computer-assisted learning remains underexplored in physiotherapy education compared to other health professions [11]. Moreover, there is limited experimental evidence evaluating the use of interactive, game-based platforms in developing practical competencies among physiotherapy students. To address this gap, the present study investigates the effectiveness of Ren’Py-based instructional materials—designed in a visual novel format—compared to conventional and hybrid teaching models. Focusing on the “medium-frequency currents” unit of an undergraduate Electrotherapy course, this study aims to assess their impact on theoretical knowledge, practical skills, cognitive load, and student motivation, all of which are essential for clinical readiness in physiotherapy education.

This study holds significance for multiple stakeholders in health professions education. For researchers, it contributes experimental evidence on the application of game-based digital platforms—such as Ren’Py—in physiotherapy education, a field where such approaches remain underexplored. For educators and clinicians, the findings offer insights into how hybrid teaching strategies may enhance skill acquisition and reduce cognitive load in learners. Ultimately, by improving the effectiveness of instructional methods in physiotherapy programs, such innovations have the potential to positively impact patient care through the training of more competent and better-prepared graduates.

We hypothesized that students receiving hybrid instruction (combining Ren’Py-based digital materials with conventional teaching) would demonstrate higher practical exam performance, lower cognitive load, and greater motivation compared to students receiving only conventional or only digital instruction.

## Methods

### Participants

**Fig. 1:**
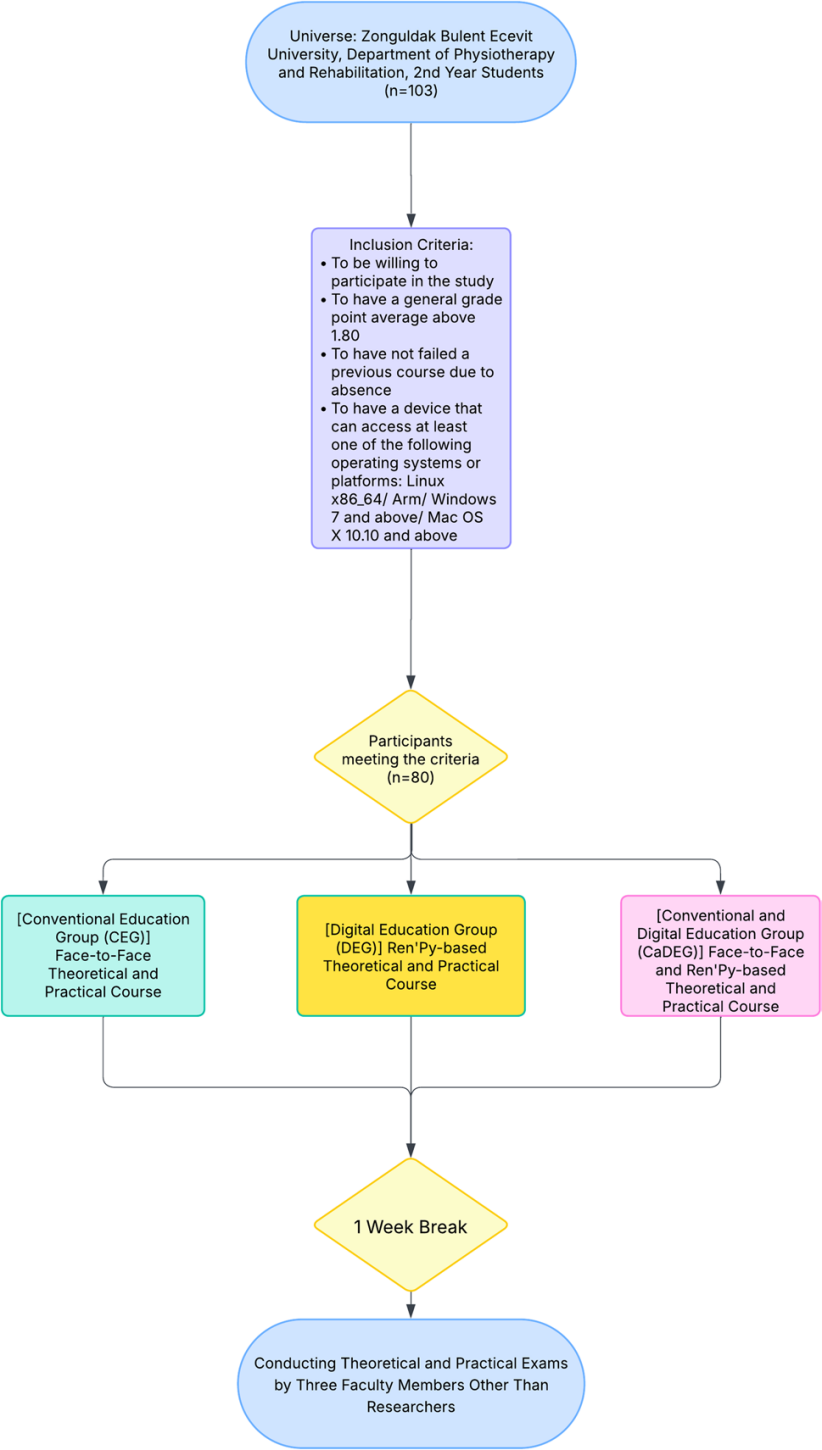
Study Flow Chart

This study was designed as a single-blind, three-arm randomized controlled trial conducted between October-December 2024 at the Department of Physiotherapy and Rehabilitation, Zonguldak Bulent Ecevit University, Türkiye. The study population consisted of 138 second-year undergraduate students enrolled in the Physiotherapy and Rehabilitation program who had not previously taken the Electrotherapy course. The sample size was calculated using the finite population correction formula for a known universe [12]. For a population of 138 eligible students, assuming an expected proportion of 0.50, a 95% confidence level (z = 1.96), and a margin of error of 0.05, the formula yielded a minimum required sample of approximately 103 students. The inclusion criteria for the study were: (1) voluntary participation, (2) a cumulative GPA above 1.80, (3) no history of course failure due to absenteeism, and (4) access to a device compatible with at least one of the following operating systems or platforms: Linux x86_64/Arm, Windows 7 or later, or Mac OS X 10.10 or later. A total of 25 students were excluded: 15 due to GPA below 1.80, 6 who declined to participate, and 2 who had previously failed a course due to absenteeism. As the study involved a healthy student population, comorbidities were not assessed and are not applicable to the study context. Finally, 80 students were included in the study. The post hoc power of the study was found to be 96.19% [13]. Participants were randomly assigned to one of three groups—Conventional Education Group (CEG), Digital Education Group (DEG), or Conventional and Digital Education Group (CaDEG)—using a random sequence generated via random.org [14]. Randomization was performed by an independent researcher who was not involved in the instruction or assessment phases. Group assignments were concealed in sealed, opaque envelopes and revealed after confirming participant eligibility. The CEG included 23 students who received conventional face-to-face instruction. The DEG consisted of 29 students who completed a fully digital learning experience using Ren’Py-based interactive visual novels. The CaDEG included 28 students who participated in a hybrid model that combined Ren’Py-based digital content with conventional course materials and teaching methods. (Fig. 1). Due to the shared classroom setting, participants were aware of their assigned instructional model. However, the individuals responsible for data collection and analysis were blinded to group allocation.

### Instructional materials and tools

In the study, the “medium frequency currents” unit taught at universities within the scope of the Electrotherapy course was preferred. There were three reasons for choosing this unit. First, medium-frequency currents are one of the widely used treatment modalities in the field of physiotherapy and rehabilitation, yielding effective results in clinical practice. This unit covers an important topic that provides students with both theoretical knowledge and practical skills. Additionally, the use of medium-frequency currents provides a foundation for students to accurately learn physiotherapy techniques and the proper use of related devices. Therefore, mastering this topic establishes a core knowledge base that supports students’ success in clinical practice. Second, the understanding and application of medium-frequency currents are considered to require a visual and interactive learning experience for students. Integrating an interactive learning platform based on Ren’Py with this unit may enable students to more effectively comprehend the characteristics of the currents, their therapeutic purposes, and their applications. In this way, it can contribute to the development of both the knowledge and practical skills of students. Finally, learning about medium-frequency currents can serve as a focal point that strengthens physiotherapy students’ knowledge and skills related to therapeutic applications. It is believed that the selection of this unit will assist students in making informed decisions when choosing appropriate treatment methods in professional practice.

1. Conventional Instructional Material: The course content was standardized using materials from the “CK4Stim—Clinical Key for Electrical Stimulation in Physiotherapy and Rehabilitation” project (CK4Stim, Access Date: October 2024). The CK4Stim project, supported by the European Union and involving multiple projects partners, aims to develop a common language for the use of electrical stimulation in physiotherapy and rehabilitation education in accordance with European standards [15]. Its target audience includes undergraduate, graduate, and doctoral students, as well as academic staff, physiotherapists, supervisors, researchers, and national and international physiotherapy associations. Written permission for the use of the content was obtained from the project coordinator. In addition, the book titled “Electrotherapy and Physical Modalities: Accompanied by Evidence and Facts” edited by Razak Özdinçler [16] was also used in the preparation of the course content.
2. Digital Instructional Material: A lesson package containing a story in an interactive visual novel format created using Ren’Py. This package was designed by the researchers to include the theoretical and practical contents of the medium frequency currents unit, again using the content of the CK4Stım project. The design took approximately 45 days. In order to check whether the students participated in this material, they were connected to Google surveys from the link that appeared on the screen at the end of the lesson and were asked to fill out the surveys within the scope of the study.

### Study flow

The instructional period for all groups was conducted over the course of one week. The Conventional Education Group (CEG) received a two-hour in-person theoretical lecture followed by a two-hour in-person practical laboratory session. The Digital Education Group (DEG) was given access to a Ren’Py-based interactive digital module via email, which they were instructed to complete asynchronously within the same one-week instructional period. Completion was verified via embedded Google Forms linked at the end of the module. The Conventional and Digital Education Group (CaDEG) received both the face-to-face sessions (as per CEG) and the full digital module (as per DEG), completed within the same week.

To assess knowledge retention, all participants underwent a one-week break following the instructional period. A one-week interval was selected to assess short-term knowledge retention, as this duration has been shown to effectively capture early consolidation and recall performance [17]. The theoretical and practical exams were then administered in a single session after this interval. All assessments were evaluated by a jury of at least three faculty members with doctoral degrees who were independent of the research team and blinded to group assignment.

### Data collection tools

1. Demographics Recording Form: It is a short form containing the participants’ age, gender and weighted grade point average information.
2. Theoretical and Practical Exams: These exams were conducted to measure the knowledge and skill levels of the students. The questions created by the researchers were also evaluated by the practical exam jury. The theoretical exam consisted of 20 multiple choice questions. The practical exam consisted of 16 questions and the jury voted “Sufficient”, “Partial”, “Insufficient” for each practical item. Both the theoretical and practical exams were evaluated out of 100. Total practical scores were determined by taking the arithmetic average of the evaluation scores of the three juries.
3. Cognitive Load Scale: It is a 9-point assessment scale developed by Paas and Van [18] to measure cognitive load. It was used to assess the mental load felt by students while working with educational materials. The Turkish validation study of the scale was conducted by Kılıç & Karadeniz [19].
4. Attitude Scale Towards Physiotherapy Profession (ASTPP): The Attitude Scale Towards Physiotherapy Profession developed by Turhan [20] is a valid and reliable tool to assess the attitudes of physiotherapists towards their profession. This scale consists of 35 items and is divided into three sub-dimensions: professional satisfaction, qualifications required by the profession, and general concerns about the profession. Each item is evaluated by responding to a five-point Likert-type scale. While 33 items on the scale are scored positively, two items are subject to reverse scoring. The lowest possible score from the scale is 35, and the highest is 175. A high score indicates that physiotherapists have positive attitudes towards their profession.
5. Instructional Materials Motivation Survey (IMMS): This scale, developed by Keller [21], measures students’ motivation levels towards educational materials. The Turkish validation of the scale was carried out by Dinçer & Doğanay [22] and it was reported that the survey was valid and reliable. This scale, which is a five-point Likert type, consists of 33 items. The highest score that can be obtained from the survey is 165, the lowest score is 33. As the score increases, motivation towards the instructional materials increases.

### Statistical analysis

The data obtained were statistically analysed using the SPSS 27.0 software package. For categorical data, frequencies and percentages were reported, while means and standard deviations were provided for quantitative data. The Shapiro-Wilk test was conducted to assess the distribution characteristics of the data. For normally distributed data, the parametric ANOVA test was applied, whereas the non-parametric Kruskal–Wallis test was used for data that did not follow a normal distribution. The chi-square test was employed for the comparison of categorical variables. Post hoc analyses were conducted using the Bonferroni test for normally distributed data and the Dunn-Bonferroni test for non-normally distributed data. A significance level of *p* < 0.05 was considered statistically significant for all findings. Baseline group homogeneity was assessed using one-way ANOVA and chi-square tests for demographic variables.

## Results

Table 1 presents the demographic characteristics of the participants. The groups were similar in terms of gender distribution (*p* = 0.975, Chi-square value: 0.05), age (CI (Confidence Interval) 95%= 19.99 to 20.58, *p* = 0.459), and grade point average (GPA, CI(95%) = 2.31 to 2.48, *p* = 0.881) (*p* > 0.05).

**Table 1:** Demographic characteristics of participants

Variables		CEG (*n*. %. X ± SD)	DEG (*n*. %. X ± SD)	CaDEG (*n*. %. X ± SD)	CI (95%)		*p*
					Lower	Upper	
Gender	Female	17 (%73.9)	21 (%72.4)	21 (%75.0)			0.975
	Male	6 (%26,1)	8 (%27.6)	7 (%25.0)			
Age		20.08 ± 1.16	20.37 ± 1.44	20.35 ± 1.33	19.99	20.58	0.694
GPA		2.43 ± 0.37	2.37 ± 0.36	2.39 ± 0.39	2.31	2.48	0.881

CEG: Conventional Education Group, DEG: Digital Education Group, CaDEG: Conventional and Digital Education Group, X: Mean, SD: Standard Deviation, n: number, %: Percent, CI: Confidence Interval

**Fig. 2:**
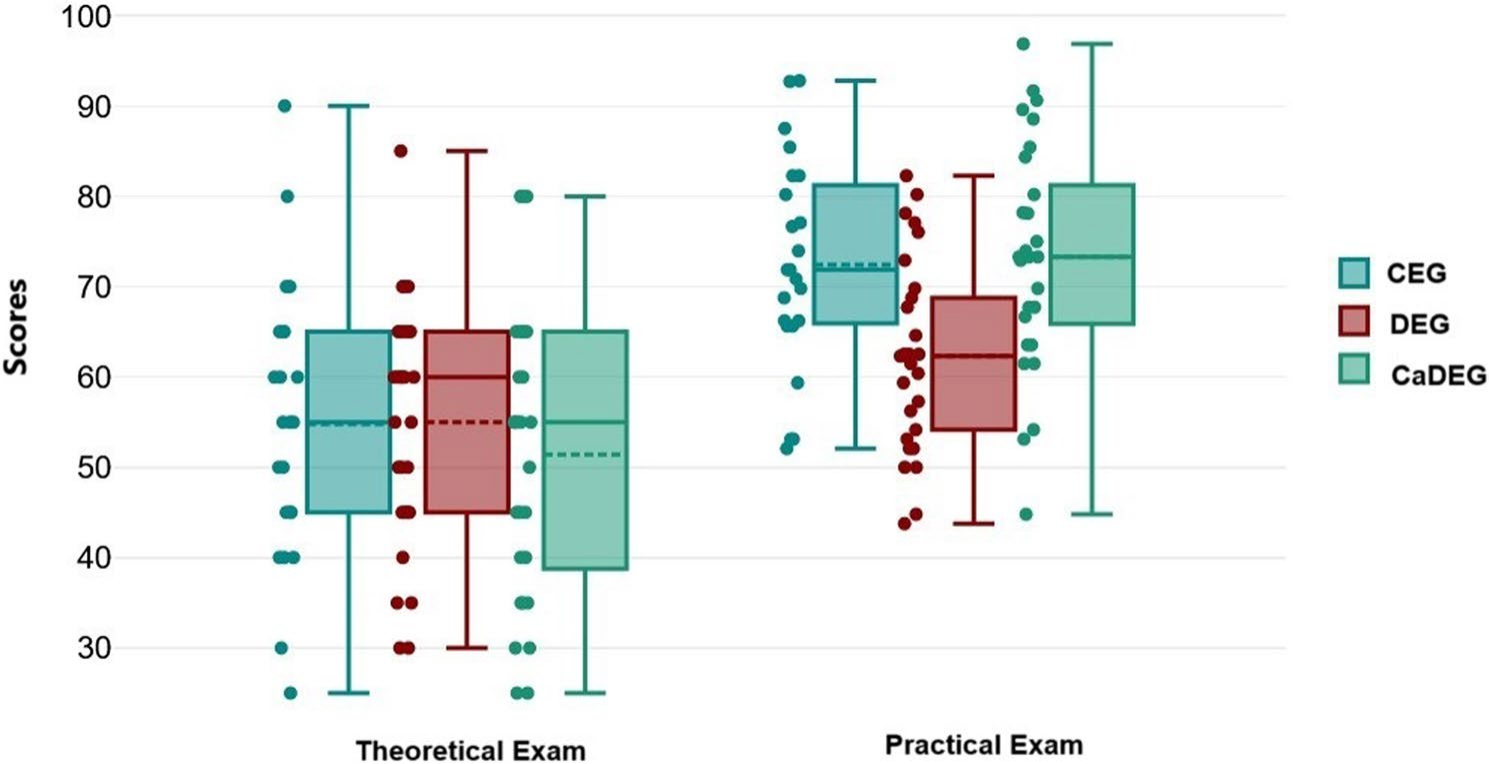
Comparison of Theoretical and Practical Exam Scores of Groups

Figure 2 shows the comparison of theoretical and practical exam scores across the groups. The theoretical exam scores were similar among all groups (degrees of freedom (df) = 2; mean square (MS) = 110.209; F = 0.487; CI(95%) = 0.00 to 0.07; *p* = 0.616). However, significant differences were observed in the practical exam scores between the groups (df = 2; MS = 1045.020; F = 7.823; CI(95%) = 0.03 to 0.30; *p* < 0.001). The results of the post hoc analysis, which compared practical exam scores between pairs of groups, are presented in Table 2. The practical exam scores of the CaDEG and CEG groups were similar (CI (95%)=−7.06 to 8.86, *p* = 1.000). Both the CEG and CaDEG groups had significantly higher practical exam scores compared to the DEG group (CI (95%) = 2.22 to 18.01, *p* = 0.007 and CI (95%)=−18.51 to −3.52, *p* = 0.002, respectively).

**Table 2:** Posthoc analysis of practical exam scores

Variables	Groups		Mean Differences	p	CI (95%)			
					Lower			Upper
Practical Exam	CaDEG	CEG	0.90	1.000	−7.06			8.86
		DEG	11.01^*^	0.002*	3.52			18.51
	CEG	CaDEG	−0.90	1.000	−8.86			7.06
		DEG	10.11^*^	0.007*	2.22			18.01
	DEG	CaDEG	−11.01^*^	0.002*	−18.51			−3.52
		CEG	−10.11^*^	0.007*	−18.01			−2.22
Cognitive Load	CaDEG	CEG	−0.98	0.020*	−2.04			0.09
		DEG	0.08	0.956	−0.93			1.08
	CEG	CaDEG	0.98	0.020*	−0.09			2.04
		DEG	1.06	0.016*	−0.01			2.11
	DEG	CaDEG	−0.08	0.956	−1.08			0.93
		CEG	−1.06	0.016*	−2.11			0.01
IMMS	CaDEG	CEG	16.04	0.022*		1.77	30.32	
		DEG	9.19	0.294		−4.24	22.62	
	CEG	CaDEG	−16.04	0.022*		−30.32	−1.77	
		DEG	−6.85	0.719		−21.01	7.30	
	DEG	CaDEG	−9.19	0.294		−22.62	4.24	
		CEG	6.85	0.719		−7.30	21.01	

CEG: Conventional Education Group, DEG: Digital Education Group, CaDEG: Conventional and Digital Education Group, IMMS: Instructional Materials Motivation Survey, CI: Confidence Interval, *p < 0.05

Table 3 shows the pre- and post-education scores of the ASTPP and the differences between post- and pre-education scores, comparing both between groups and within groups. There were no significant differences between the groups in pre-education, post-education, or post-pre education difference ASTPP scores (CI(95%) = 130.13 to 136.16, *p* = 0.858; CI(95%) = 131.91 to 137.08, *p* = 0.511; CI(95%)=−1.27 to 3.97, *p* = 0.852, respectively). Similarly, within-group comparisons of pre- and post-training ASTPP scores showed no significant difference in any of the CEG, DEG and CaDEG groups (CI(95%)=−4.59 to 3.63, *p* = 0.987; CI(95%)=−7.31 to 5.10, *p* = 0.335; CI(95%)=−5.22 to 0.57, *p* = 0.075, respectively).

**Table 3:** Comparison of ASTPP scores before, after and after-training difference scores before training

ASTPP		CEG (X ± SD)	DEG (X ± SD)	CaDEG (X ± SD)	df	Mean Square	F	CI (95%)		*p*
								Lower	Upper	
Pre-education		132,91 ± 14,28	132,27 ± 14,60	134,25 ± 12,12	2	28,665	0,153	130.13	136.16	0.858
Post-education		133,39 ± 12,55	133,37 ± 11,98	136,57 ± 10,55	2	92,419	0,678	131.91	137.08	0.511
z		−0.016	−0.964	−1.780						
p		0.987	0.335	0.075						
CI (95%)	Lower	−4.59	−7.31	−5.22						
	Upper	3.63	5.10	0.57						
(Post-education)- (Pre-education)		2,32 ± 7,47	0,47 ± 9,51	1,10 ± 16,32	2	22,832	0,160	−1.27	3.97	0.852

CEG: Conventional Education Group, DEG: Digital Education Group, CaDEG: Conventional and Digital Education Group, ASTPP: Attitude Scale Towards Physiotherapy Profession, df: degree of freedom, X: Mean, SD: Standard Deviation

**Fig. 3:**
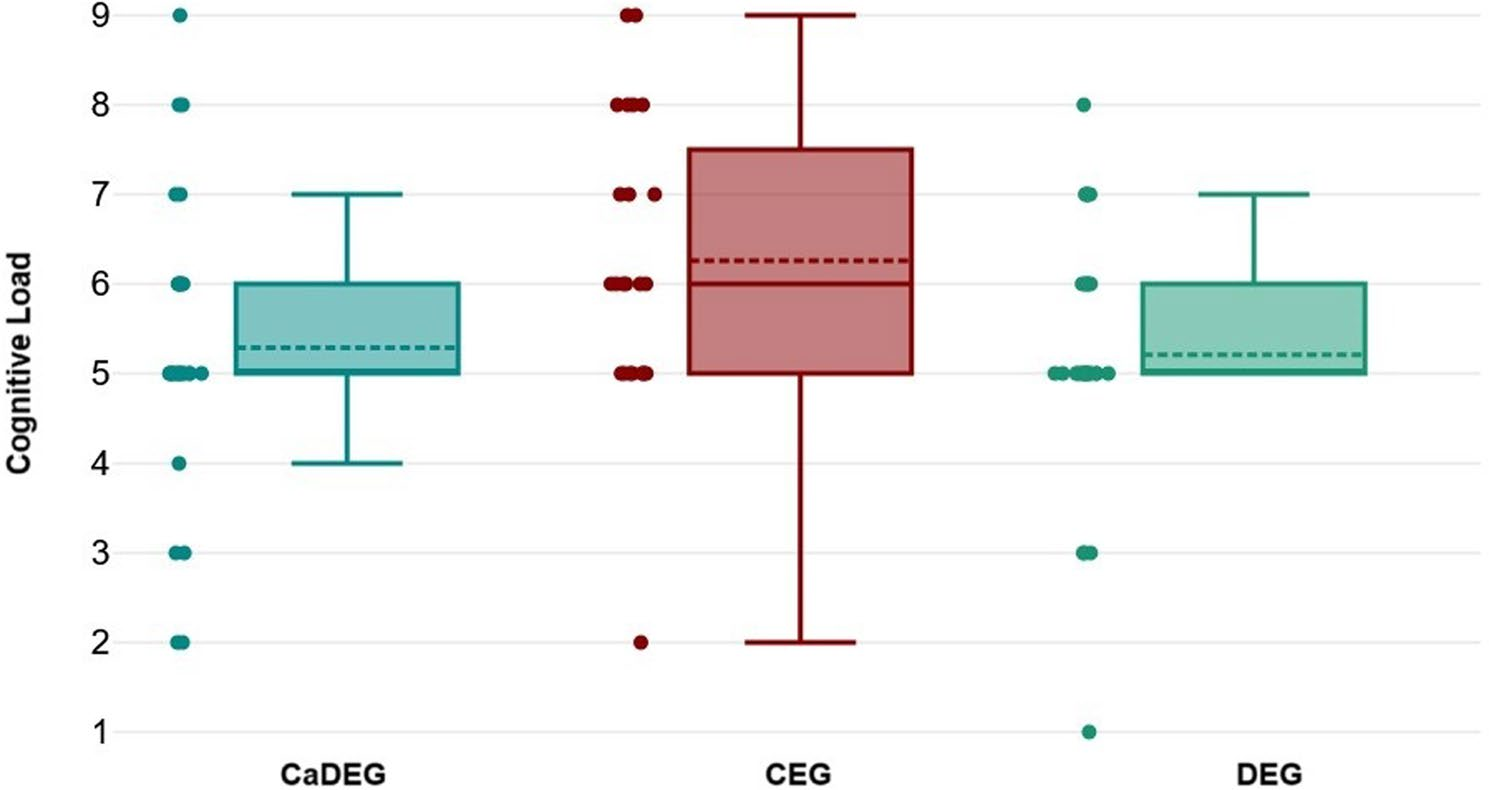
Comparison of Cognitive Load between groups

Figure 3 shows the comparison of cognitive load assessed after the training across the groups. The findings indicated significant differences in post-education cognitive load between the groups (df = 2, Kruskal-Wallis test statistic = 7.218, CI(95%) = 0.00 to 0.09; *p* = 0.027). The results of the post hoc analysis, which compared cognitive load scores between pairs of groups, are presented in Table 2. The CEG group reported significantly higher cognitive load compared to both the CaDEG and DEG groups (CI(95%)=−2.04 to 0.09, *p* = 0.020; CI(95%)=−0.01 to 2.11, *p* = 0.016, respectively). The cognitive load reported by the CaDEG group was similar to that of the DEG group (CI(95%)=−0.93 to 1.08, *p* = 0.956).

**Fig. 4:**
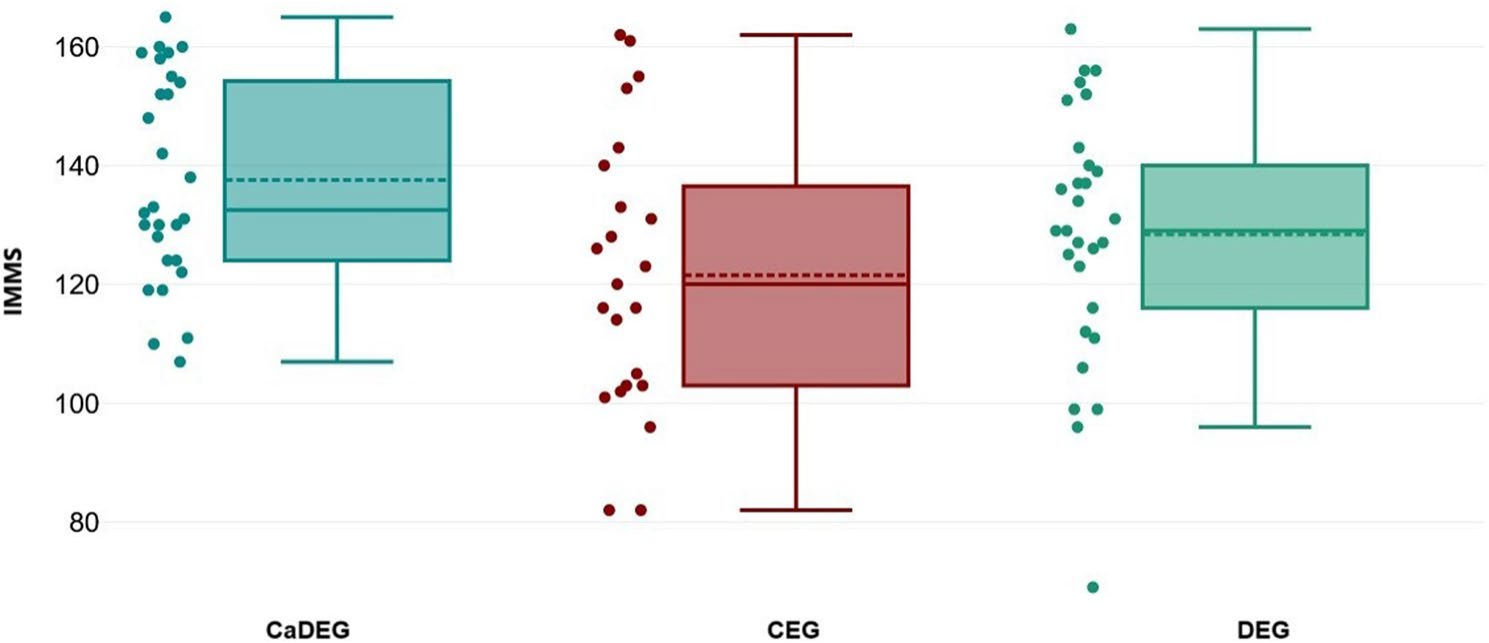
Comparison of IMMS Scores between groups

Figure 4 presents the comparison of IMMS scores assessed after the training across the groups. The findings indicated significant differences in post-education IMMS scores between the groups (df = 2; MS = 1661.663; F = 3.870; CI(95%) = 0.00 to 0.21; *p* = 0.025). The results of the post hoc analysis, which compared IMMS scores between pairs of groups, are presented in Table 2. The IMMS scores of the CaDEG group were significantly higher than those of the CEG group (CI(95%) = 1.77 to 30.32, *p* = 0.022). The IMMS scores of the DEG group were similar to those of both the CaDEG and CEG groups (CI(95%)=−4.24 to 22.62, *p* = 0.294; CI(95%)=−21.01 to 7.30, *p* = 0.719).

## Discussion

This study examined the effects of conventional, digital, and hybrid instructional methods in physiotherapy education. While theoretical knowledge acquisition did not differ significantly between groups, practical exam scores were higher in groups receiving face-to-face or hybrid instruction. The hybrid group also reported lower cognitive load and greater motivation compared to the conventional group. No significant changes were observed in professional attitude scores (ASTPP).

The average scores of the groups in the theoretical exam were similar, whereas significant differences were observed in the practical exam scores between the groups. These results suggest that there is no substantial difference between instructional methods in terms of theoretical knowledge acquisition; however, face-to-face education appears to provide a significant advantage in the development of practical skills. The literature presents mixed findings on this topic. Some studies have reported that digital technology has a positive impact on enhancing students’ knowledge levels and improving their academic performance, while others have found no significant effect [23–26].

The fact that the practical exam scores of the CaDEG and CEG groups were similar suggests that face-to-face education, whether completely face-to-face or in a hybrid model, is effective in the development of practical skills. On the other hand, it has been stated that online technologies are useful in terms of physiotherapy students gaining practical skills, developing knowledge and thinking, and accessing learning resources more easily [23]. The fact that the practical exam scores of the DEG group, which received education only in the digital classroom, were significantly lower than the other groups shows that face-to-face education plays an important role in teaching practical skills. In a study conducted using the blended learning method, positive developments were observed in the students’ practical competence and general performance [27]. Similarly, in a study conducted using virtual simulations, nursing students stated that they benefited from simulations when preparing for practical exams, but they preferred to use technology together with conventional teaching methods [28]. All these results reveal that online education models in practice-based fields such as physiotherapy should be enriched with additional methods to support practical skill acquisition.

No significant difference was observed in ASTPP scores before or after the intervention across all groups. This may be due to a ceiling effect or the short intervention duration. Similar findings have been reported in studies where short-term educational strategies did not shift professional attitudes. The cognitive load reported by the CEG group was significantly higher than the CaDEG and DEG groups, whereas no statistically significant difference was found between the CaDEG and DEG groups. Instructional design is thought to play a critical role in effectively controlling cognitive load [29]. It has been reported in the literature that perceptually rich elements such as detailed visuals and interactive responses provided by learning applications contribute to the improvement of learning outcomes, while at the same time creating a certain amount of unnecessary cognitive load [30].

In online learning, the use of gamification is generally considered a method with the potential to produce motivating and enjoyable learning experiences [30], and interactive learning formats have long been assumed to increase student motivation and engagement [31]. In our study, there were significant differences between the groups in the IMMS scores obtained after the training. The IMMS scores of the CaDEG group were found to be significantly higher than the CEG group. This suggests that the hybrid approach may be effective in increasing students’ motivation towards the teaching materials. On the other hand, the IMMS scores of the DEG group were similar when compared to the CaDEG and CEG groups. This indicates that the digital education model does not significantly increase students’ motivation towards the teaching materials when applied alone; however, this motivation may increase when supported by face-to-face interaction (hybrid model).

This study compares the effects of different teaching models (face-to-face, digital and hybrid) in physiotherapy education and provides valuable data on various learning outcomes such as theoretical and practical performance, cognitive load and motivation towards the teaching material. Its strengths include systematic comparison of various teaching models as well as detailed analyses of critical measures such as practical skills and cognitive load. The superiority of face-to-face and hybrid approaches over digital methods, especially in practical skill performance, emphasizes the importance of face-to-face interaction in practical education processes. These results suggest that integrating digital tools into existing instruction may enhance student engagement and practical performance.

On the other hand, the limitations of the study include the relatively small sample size and short intervention period, which may affect the generalizability of the results. Although all participants were taking the course for the first time, individual factors such as differences in digital literacy, learning styles, or motivation were not assessed at baseline and may have influenced how students interacted with the instructional materials. The short duration of the instructional period and absence of long-term follow-up may limit the interpretation of sustained learning outcomes and attitudinal changes. Additionally, occasional software and hardware problems were encountered and resolved with the researchers’ assistance; however, this proved that digital implementations such as these create a certain technical overhead that needs to be accounted for. In the absence of such resources, the creation of the software alone could result in an inefficient educational setting.

This study was conducted as a single-blind, three-arm randomized controlled trial. Randomization was performed by an independent researcher who was not involved in the instruction or outcome assessment, ensuring procedural separation and minimizing potential bias.

## Conclusions

This study examined the effects of different teaching models on students’ learning outcomes in physiotherapy education and found significant differences in practical skill performance, cognitive load, and motivation. Face-to-face and hybrid models were more effective than digital-only instruction in supporting practical skills, likely due to the benefits of real-time feedback and applied learning. These findings underscore the continued importance of face-to-face interaction, while also highlighting the potential of digital tools as complementary resources. Research on these complementary techniques can greatly enhance educational quality and efficiency in the long term, allowing for better clinical outcomes overall. Although tools like Ren’Py were not sufficient on their own to support practical performance, they may offer added value when integrated into hybrid formats. Future research with extended instructional periods and larger samples is warranted to further explore the role of interactive software in enhancing cognitive engagement and supporting sustained learning.

## Supplementary Information

Supplementary Material 1.

Supplementary Material 2.

Supplementary Material 3.

Supplementary Material 4.

## Data Availability

The datasets used and/or analysed during the current study are available from the corresponding author on reasonable request.

## Abbreviations

CEG: Conventional Education Group
DEG: Digital Education Group
CaDEG: Conventional and Digital Education Group
ASTPP: Attitude Scale Towards Physiotherapy Profession
IMMS: Instructional Materials Motivation Survey
