# Supplementary figures and images for: Digitalization through the use of Ren’Py-Based interactive learning experiences in physiotherapy and rehabilitation education: a randomised, controlled, single-blind study
Source: BMC Med Educ. 2025 Dec 22;26:538. doi: 10.1186/s12909-025-08474-z (PMC13040823; doi:10.1186/s12909-025-08474-z)

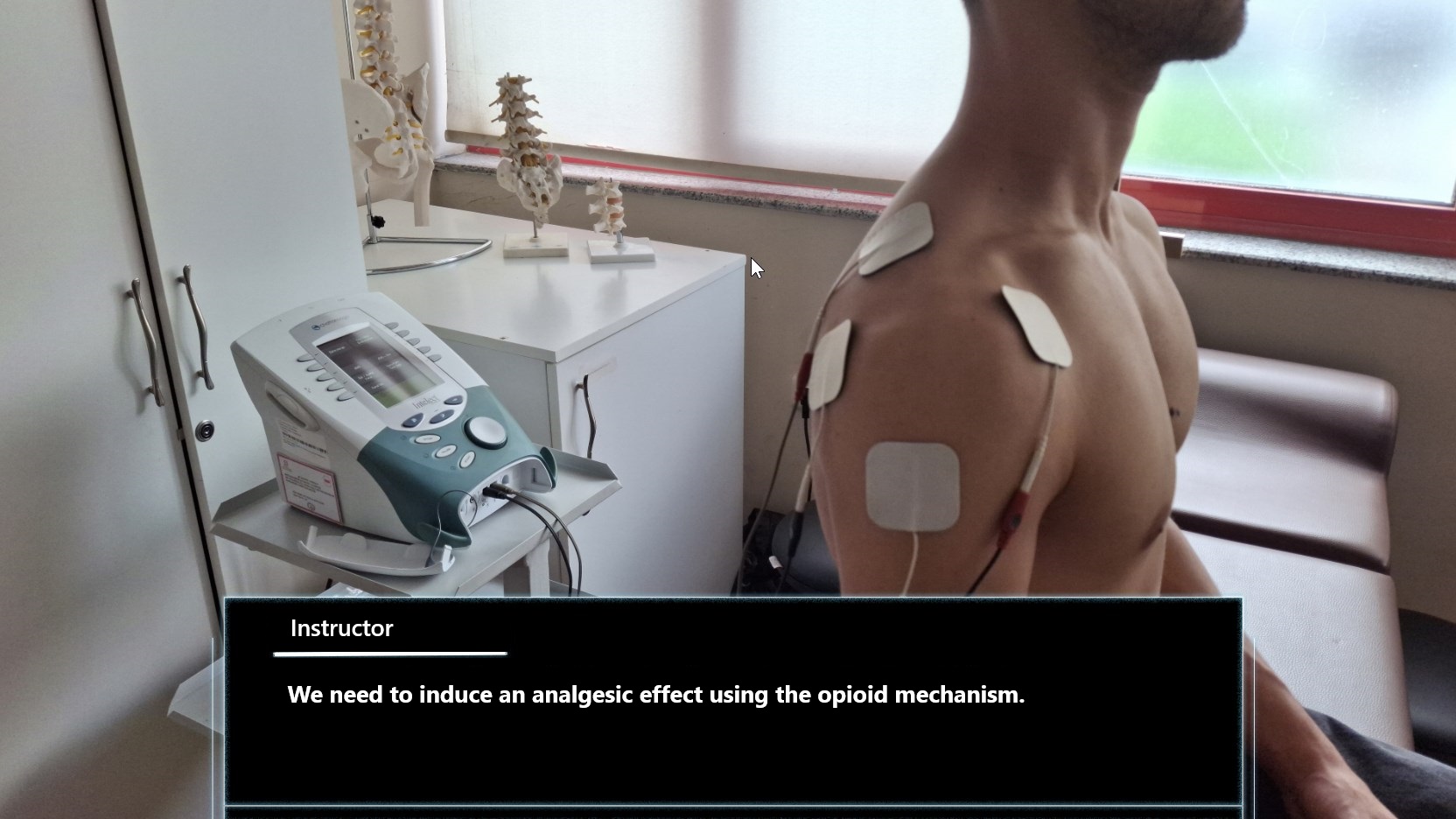

Supplement: Supplementary file 1 — Supplementary Material 1. [file 12909_2025_8474_MOESM1_ESM.jpg]

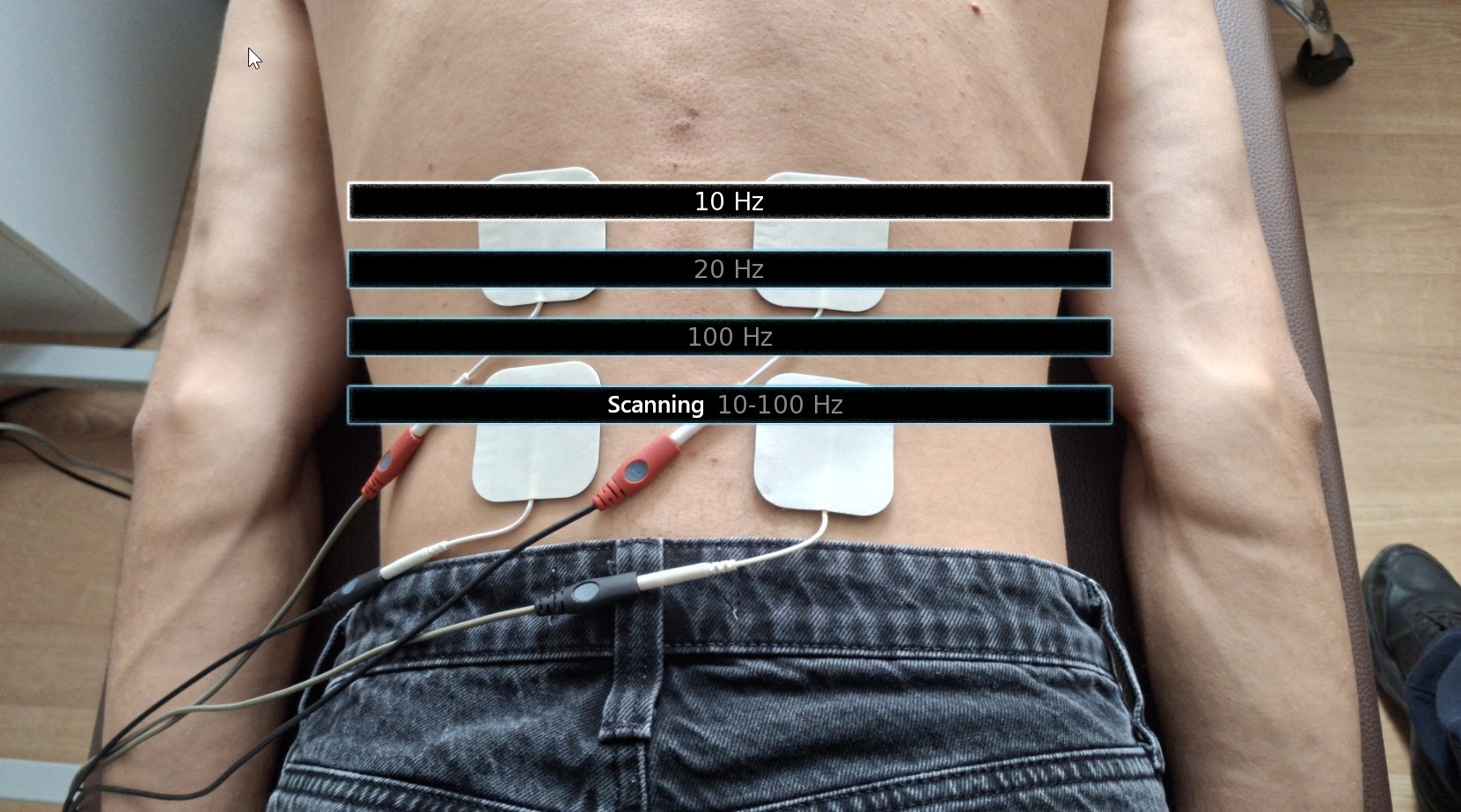

Supplement: Supplementary file 2 — Supplementary Material 2. [file 12909_2025_8474_MOESM2_ESM.jpg]

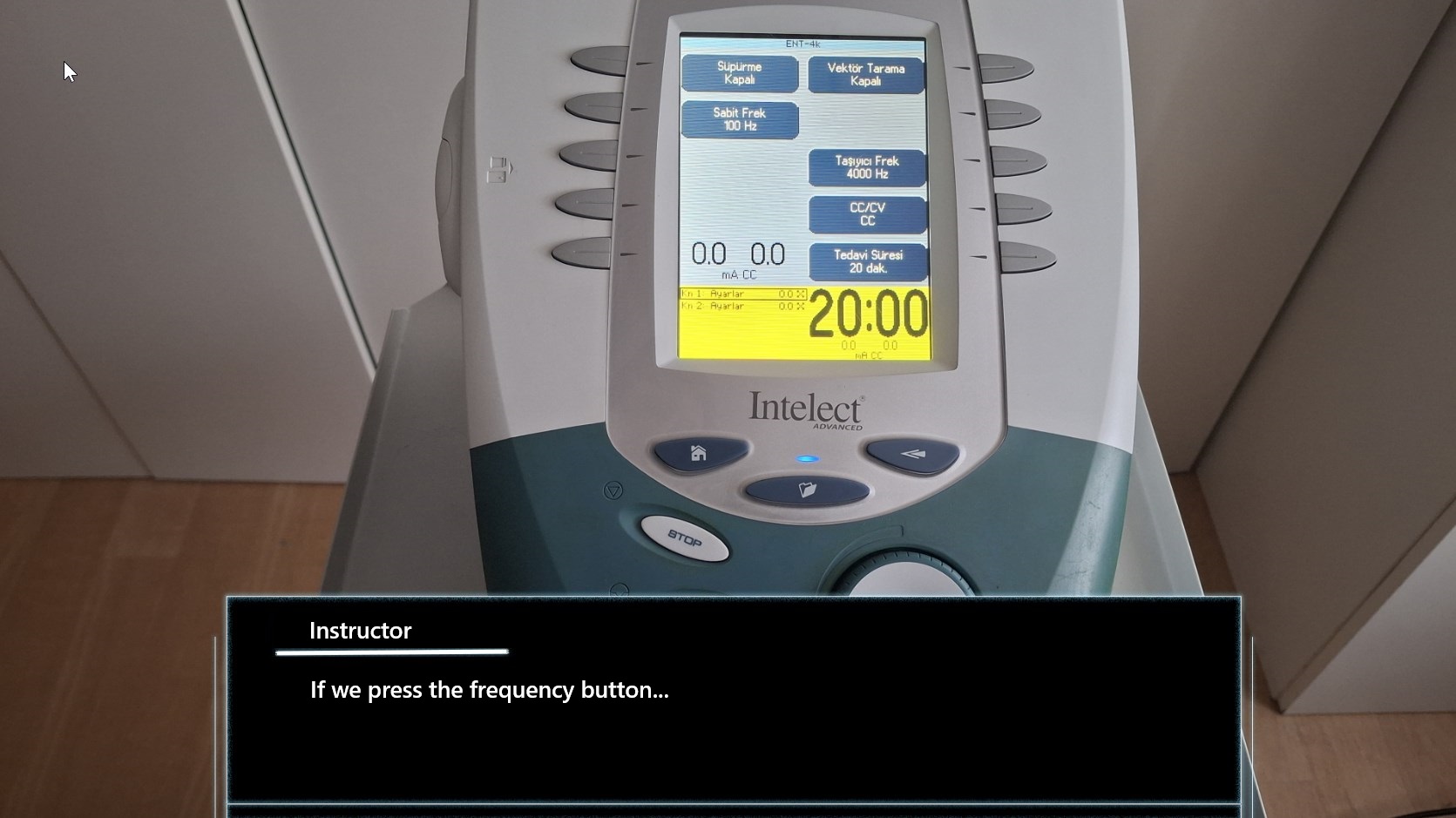

Supplement: Supplementary file 3 — Supplementary Material 3. [file 12909_2025_8474_MOESM3_ESM.jpg]

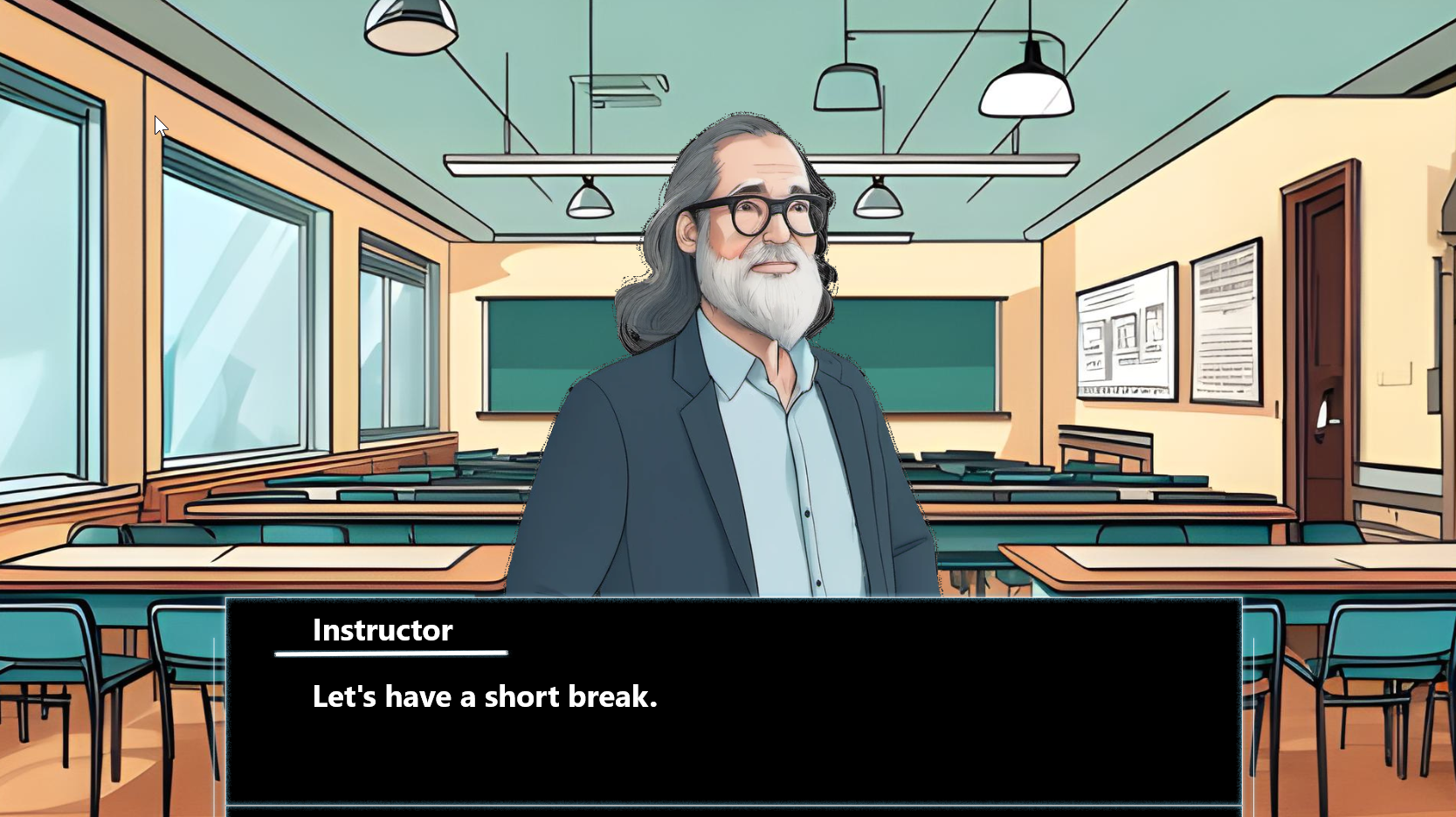

Supplement: Supplementary file 4 — Supplementary Material 4. [file 12909_2025_8474_MOESM4_ESM.png]
